# Differences in Knowledge, Attitude, and Behavior towards HIV/AIDS and Sexually Transmitted Infections between Sexually Active Foreign and Chinese Medical Students

**DOI:** 10.1155/2016/4524862

**Published:** 2016-04-19

**Authors:** Martin Kuete, Qiao Huang, Abid Rashid, Xiu Lan Ma, HongFang Yuan, Juan Pablo Escalera Antezana, Rakhmanov Yeltay, Meng Rao, Qian He, ChengLiang Xiong, HuiPing Zhang

**Affiliations:** ^1^Family Planning and Research Institute, Tongji Medical College, Huazhong University of Science and Technology, Wuhan, Hubei 430030, China; ^2^Faculty of Medicine and Biomedical Sciences, The University of Yaoundé I, P.O. Box 3011, Messa, Yaoundé, Cameroon; ^3^School of Nursing, Tongji Medical College, Huazhong University of Science and Technology, Wuhan, Hubei 430030, China; ^4^Department of Pediatrics, Tongji Hospital, Tongji Medical College, Huazhong University of Science and Technology, Wuhan, Hubei 430030, China; ^5^Department of Trauma Surgery, Tongji Medical College, Huazhong University of Science and Technology, Wuhan, Hubei 430030, China; ^6^Department of Orthopaedics, Tongji Hospital, Tongji Medical College, Huazhong University of Science and Technology, Wuhan, Hubei 430030, China; ^7^Department of Epidemiology and Biostatistics, School of Public Health, Tongji Medical College, Huazhong University of Science and Technology, Wuhan, Hubei 430030, China

## Abstract

Although the prevalence of human immunodeficiency virus (HIV) decreased in the last decade worldwide, the number of deaths due to HIV/AIDS and communicable diseases including syphilis, hepatitis, and tuberculosis had dramatically increased in developing countries. Education and behavior are incredibly important factors to prevent these diseases' spread. This study highlights the range of differences in knowledge, attitude, and behavior of 434 sexually active medical students towards HIV/AIDS and sexually transmitted infections (STIs). Because the surveyed population constitutes the forefront of healthcare providers and was originated from different area of the world, this is the first time a study sought to investigate the behavioral attitude of this group of population irrespective of the three levels of their academic and professional knowledge. Several factors including sociodemographic characteristics, sexual behavior, HIV/AIDS, and STIs related patterns play a key role in medical student attitude and behavior towards people infected with HIV/AIDS and STIs. Our findings add consistent value in prior studies which aimed to stop new infections and also imply further investigations on the management of the studied infections by medical students. The present study arouses much interest among participants and provides evidence of reinforcing medical students' education on HIV/AIDS and STIs.

## 1. Introduction

Despite the great effort made by governments and international organisations to stop new HIV infection and reduce sexually transmitted infections by 2015 [1, 2], evidences show that communicable infections continue to thrive while decimating millions of lives in developing countries [3–7]. These infections are going beyond the most vulnerable population groups worldwide including migrants. Many studies demonstrated that HIV infection is shifting from men who have sex with men (MSM) to heterosexuals especially among youth at reproduction age [8–10]. The current state is more apparent in China where local and overseas migration and economic development are rapidly growing up. Alongside of this, China is the most populous country in the world and may play a key role in efficiently managing epidemics. Recently, because of nonachievement of millennium development goals on HIV/AIDS in different regions in the world especially in developing countries, the Joint United Nations Programme on HIV/AIDS has fixed new objectives to overcome HIV infection by 2030 [11].

Looking at the paradigm to fight against HIV with the aim of providing the treatment to all infected people regardless of their biological and clinical stage [12], several other key objectives such as making antiretroviral therapy (ART) available and accessible to all patients, strengthening healthcare systems, or improving prevention programmes across general populations are also worthy. Additionally, enhancing strategic measures to encourage the overwhelming number of HIV infected unknown individuals to undergo the test, engaging newly infected people into the treatment programme and healthcare providers support, and advancement of testing, treatment, and follow-up of patients are crucial.

In the last decade, China became not only more attractive in business or political affairs but also one of the main international destinations for education, especially in the field of medicine. Many foreign students studying in Chinese universities are from countries with high prevalence of communicable diseases; it is gainful and invaluable to have extensive knowledge in HIV/AIDS and STIs for practical issues when returning back home after graduation. Then, several studies revealed that barriers including lack of adequate knowledge, misconceptions, negative feeling, refusal, or even discrimination were found among healthcare professional in providing medical care and monitoring HIV/AIDS patients [13–19]. Therefore, to end HIV/AIDS epidemics in the further years, medical students are the workforce of health system and should be provided with well training in HIV/AIDS and sexually transmitted diseases to be able to provide holistic care regarding these infections which are predominant in developing world. In this study, we hypothesized that differences existed in knowledge, attitude, and behavior related to HIV/AIDS and sexually transmitted infections among foreign and local medical students trained in China. Therefore, we aimed to explore and analyze the differences existing in knowledge, attitude, and behavior towards HIV/AIDS and STIs among foreign and local medical students in Chinese university.

## 2. Methods

### 2.1. Study Design

The authors designed a cross-sectional study using validated self-administered questionnaire. We focused on the rationale of this study to compare and show differences existing between foreigner and Chinese medical students' knowledge, attitude, and behavior (KAB) towards HIV/STIs infected persons. The survey tool was firstly designed in English for foreign students then translated into Chinese language. Both English and Chinese versions were discussed and pilot-studied before the final usage.

### 2.2. Setting, Location, and Period of the Study

This study was conducted in Tongji Medical College affiliated to Huazhong University of Science and Technology (HUST) located in Wuhan, the capital city of Hubei province known as the Chinese education city. According to the latest Chinese ranking universities, Tongji Medical College belongs to the top 1% of Chinese medical schools and is the mostly reputed for its numerous medical programs taught in English. The number of foreigners enrolled for a degree programme during the last 5 years has significantly increased to about 1500 international students annually [20, 21]. Participants were recruited during the normal weekends from March 01 to June 14, 2015.

### 2.3. Participants Study Size and Eligibility

Total of 434 medical students (MBBS/Bachelor, Master, and Doctor) were randomly selected among students regularly registered and living in students' campus apartments. 157 foreign students enrolled in English medical degree programme and 277 Chinese medical students all sexually active were included in this study (student who had have or are currently engaged with a sexual partner).

### 2.4. Sources and Methods of Selection of Participants

All students were previously informed of educational based characteristics of the survey through posters announcement and presurvey activities formed to inform and discuss with respondents publicly across the school campus. During this phase, investigators covered approximately 98% of study population. The final step consisted of using “*student door-by-door strategy*” which consisted of randomly selecting student's room from all apartments in the campus residency and inviting student to participate. To ensure the confidentiality and increase participation rate, student was asked to drop the anonymous questionnaire filled in a box placed at the entry of apartment. All students enrolled in nondegree programme and nonsexually active were excluded. Participation rate was estimated to be 65% for foreigners and 78% of surveyed local students.

### 2.5. Questionnaire and Measurement

#### 2.5.1. Participants' Baseline Characteristics

The following variables were gathered: gender, age, education level, marital status, region of origin, sexual orientation, number of sex partners, frequency of condom use and enjoyment during sexual intercourse, history of HIV and STIs testing, and vaccination for curable infections (syphilis, hepatitis B and hepatitis C, and tuberculosis).

#### 2.5.2. HIV/AIDS Knowledge and Sources of Information

The variables were measured through a series of 7 yes or no questions on HIV/AIDS cause, progression, and prevention; the main sources of information included 8 items while the assessment of HIV risk factors was subdivided into the route of transmission containing right and wrong responses (16 items), high risk individuals (8 items), and risk factor increasing HIV transmission (7 items). In addition, 3 other questions were related to previous extracurricular training in HIV and STIs, student belief of sufficient knowledge about studied infections (HIV/AIDS, syphilis, hepatitis, and tuberculosis), and student willingness to know more about HIV/AIDS.

#### 2.5.3. Attitude and Behavior

Variables describing student attitude and behavior comprised 21 questions mainly on sexual behavior and condom use, HIV and STIs testing, their attitude vis-à-vis friend/classmate infected with HIV, and students' opinions related to the support of government and NGOs towards HIV/AIDS patients. We also assessed student's beliefs regarding HIV/AIDS comparing to other STIs and tuberculosis and moreover the relationship between HIV infection and ethnicity and the current development of China.

#### 2.5.4. Discrimination and Support of Medical Students towards HIV/AIDS Individuals

Variables describing the discrimination and support of students towards HIV/AIDS patients derived from 13 items of the questionnaire which included both positively and negatively framed questions.

### 2.6. Statistical Methods

Data entry was done using Epidata version 3.5 and all analyses were conducted with SPSS version 18.0. In short, we used numbers and percentages to present most of data while *P*_50_ (*P*_25_–*P*_75_) was use for nonnormal continuous variables; the Mann-Whitney *U* test (*Z*-value) for continuous variables was conducted to compare measurements between Chinese and foreign students. Categorical variables were compared using *χ* ^2^ (for comparing proportions) tests. Where possible when assessing the knowledge, attitude, and behavior, we assigned 1 for every positive response and 0 for wrong answer. For brevity and clarity, we reported only significant factors influencing discrimination and support in multivariate logistic regression analyses. Odds ratio (OR) with 95% of confident interval (CI) was calculated to fit association levels between students' characteristics and discrimination or support of HIV/AIDS individuals. All tests were two-tailed and results were considered significant at *p* < 0.05.

### 2.7. Ethics Statement

Ethical clearance was previously obtained from the institutional review board of Tongji Medical College of Huazhong University of Science and Technology under authorization number* IORG S115*. Moreover, all participants provided a written informed consent attached with the questionnaire at the time of the survey.

## 3. Results

### 3.1. Students' Baseline Characteristics

Data summarized in Table 1 showed that no statistical difference existed between these sexually active students regarding their education level and condom use during sexual intercourse. However, many patterns including gender, marital status, sexual orientation, HIV testing, and screening for other infections studied (syphilis, hepatitis B and hepatitis C, and tuberculosis) significantly varied between foreign and Chinese medical students.

### 3.2. Knowledge and Sources of Information Related to HIV/AIDS

Despite the fact that no difference was found in participants' education level, this study revealed a huge difference in HIV/AIDS knowledge between Chinese and foreign students (see Table 2). Overall, Chinese students were more knowledgeable compared with their counterparts in many factors. For instance, only 49.68% of foreign students compared to 95.67% of Chinese provided the right answer related to the nature of pathogen. In addition, 34.39% of foreigners versus 90.97% of local students agreed that condom makes sexual intercourse safe in HIV acquisition. Contrary to Chinese students, whose source of information was mainly from school education, foreign medical students were more likely to use Internet; the majority of students reported an extracurricular training in HIV/STIs and almost all had wished to know more about HIV/AIDS.

**Figure 1:**
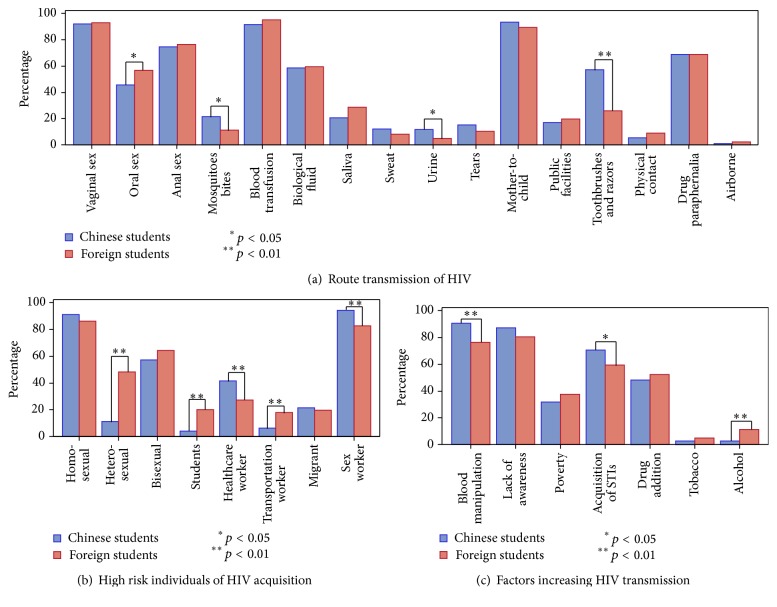
Distribution of knowledge related to HIV transmission routes, high risk individuals of HIV acquisition, and factors increasing HIV transmission.

Confusions about route transmission and risk factors of HIV transmission were found among the two groups. Differences presented in Figure 1 showed that medical student knowledge remained inconsistent. Many students cited oral sex, mosquitoes bites, saliva, sweat, urine, tears, public health facilities, and physical contact as route of transmission of HIV (Figure 1(a)). Many also perceived students, healthcare providers, or migrants as high risk populations of HIV acquisition (Figure 1(b)); meanwhile despite the conventional known factors (blood manipulation, acquisition of STI, illicit drug addiction, etc.) which may increase HIV transmission, poverty or tobacco consumption was also enumerated to increasing HIV transmission (Figure 1(c)).

### 3.3. Attitude and Behavior of Medical Students towards People Infected with HIV/AIDS

Results given in Table 3 showed both difference and similitude of medical students' attitude and behavior. 45.49% of Chinese students compared to 35.67% of foreigners opted to avoid condom usage after HIV testing of partners while 16.97% versus 22.93% agreed to have unprotected sex after mutual decision without HIV testing. Regarding the medical students' attitude vis-à-vis their infected counterparts, the two groups of study population may react so differently and, however, 28.88% of Chinese students would prefer to keep away from the infected individual compared to 7.64% of foreigners. Similarly, participants exhibited the same attitude towards other people living with HIV/AIDS. Though the majority from each group expressed positive attitude of healthcare providers to support patients with HIV, only fewer admitted an intimate relationship between HIV infected individual and noninfected person. Furthermore, the large majority of students in the two groups have shown positive attitude of supporting HIV/AIDS patients in counseling, education, promoting the behavior change of population, or acting as peer educator; however, over 25% refused promoting patient rights and considered HIV infection highly dangerous comparing to syphilis, hepatitis B and hepatitis C, or tuberculosis.

### 3.4. Multiple Logistic Regression Analysis of Discrimination and Support of Medical Students towards People with HIV/AIDS and STIs Infected

From the multiple logistic regression analysis, results presented in Table 4 provided associations between medical students' background and the negative or positive behavior vis-à-vis PLWHA. Age (OR: 0.09, 95% CI: 0.03–0.27), number of sexual partners (OR: 1.85, 95% CI: 1.10–3.12), recent HIV testing (OR: 2.45, 95% CI: 0.91–6.60), perceived medical students as high risk population (OR: 2.47, 95% CI: 1.01–6.03), and being informed through books and newspapers from HIV/AIDS (OR: 1.86, 95% CI: 1.10–3.17) were associated with the discrimination of people infected with HIV by medical students; and, however, Chinese medical students seemed to tolerate living with people infected with HIV/AIDS compared to foreign students (OR: 0.09, 95% CI: 0.03–0.27).

Concerning the support to HIV infected individuals, several factors including students' origin (OR: 3.78; 95% CI: 1.41–10.13), high level of knowledge about HIV transmission (OR: 1.91; 95% CI: 1.1–3.31), and manifestations (OR: 9.47; 95% CI: 3.7–24.27), being bisexual (OR: 1.81; 95% CI: 1.1–2.97), and vulnerability factors such as lack of knowledge about HIV/AIDS (OR: 1.93; 95% CI: 1.02–3.65) or poverty (OR: 2.2; 95% CI: 1.28–3.79), condom usage (OR: 2.62; 95% CI: 1.32–5.24), source of information (OR: 2.07; 95% CI: 1.02–4.22), and the willingness to know more about HIV/AIDS (OR: 3.03; 95% CI: 1.66–5.55) were associated with the support that students may provide to HIV/AIDS individuals; however, never having been tested (OR: 0.36; 95% CI: 0.18–0.73) or vaccinated for hepatitis B (OR: 0.5; 95%: CI 0.27–0.94) and knowing that STIs acquisition increase HIV transmission (OR: 0.39; 95% CI: 0.22–0.68) could negatively affect students' behavior vis-à-vis HIV infected individuals.

## 4. Discussion

### 4.1. Participants' Background, HIV/AIDS, and STIs Knowledge

Human immunodeficiency virus and acquired immunodeficiency syndrome (HIV/AIDS) are known as a major public health concern worldwide. The constant assessment of healthcare provider knowledge and behavior would always contribute to overcoming the infection. No previous study has assessed and compared medical student's knowledge and behavior towards HIV/AIDS and associated sexually transmitted infections among local students and their foreign counterparts in China. Whether differences were found across countries in students' knowledge, attitudes, and behavior [22–24], medical students are the milestone of healthcare providers. No matter where they are from and were trained, they are in the forefront of medical care and may play a key role in different health areas as well as in globalization health. Though all participants were sexually active, no difference regarding education level existed among the survey population. However, major differences were observed between the two groups. Chinese students were slightly older than foreigners and many were currently engaged with sexual partner. This result may be obviously due to the stability of participant that provides consistent reasons to feel close and project common goals between Chinese students.

This study revealed high rate of homosexuals among Chinese students compared with foreigners. Despite the fact that homosexuality is contrary to Chinese values and not yet accepted, some Chinese young men and women are sexually going with the same sex partner [9]. Of note, HIV transmission is mainly drawn by MSM in China [5]. However, recent studies reported that HIV transmission is shifting into heterosexual populations [2, 8, 10, 25]. Foreign students were more likely to have more than one sexual partner with inconsistent condom use certainly because they feel uncomfortable with condoms. Despite the missing information on casual sex intercourses of participants, we speculate that instability previously described may lead foreigners to experience more occasional relations contrary to Chinese students. Results of HIV, syphilis, hepatitis, and tuberculosis testing history showed that almost all foreigners had been tested for HIV while only fewer Chinese medical students were aware of their HIV status. Similar observation occurred when assessing syphilis, hepatitis, and tuberculosis screening history. The difference observed is certainly resulting from the Chinese government requirements for foreigners to have physical examination for communicable diseases including HIV/AIDS, syphilis, hepatitis, and tuberculosis before coming to China or applying for temporary residency. Despite the medical status of participants, findings revealed independently of education level and region of origin that student's sexual behavior is questionable and merits changes regarding their risk exposure to HIV and STIs.

Despite the greater effort made by international organisations, nongovernmental organisations, and the majority of governmental institutions in developing world to inform and educate about communicable diseases, several misconceptions are persistent among students including medical students on HIV/AIDS and STIs [18, 26, 27]. Independent of their education level and region of origin, this study showed a huge gap in participants' knowledge. For example, almost all Chinese students provided the right answer when identifying the nature of AIDS pathogen compared to less than half of foreigners; concerning the earliest related time for HIV positive testing after exposure only forty-one per cent of foreigners identified the right answer compared to twenty-seven per cent of Chinese students. More than eighteen years after Cook et al. found discrepancies in HIV knowledge among medical students and suggested innovative methods to improve students' awareness on HIV [14], confusion and misconception still remain. In this study, more than twenty per cent of medical students reported mosquitoes bites, tears, saliva, and public facilities as transmission route while about forty per cent cited poverty as factor increasing HIV transmission. The education level and extracurricular training on HIV/AIDS of participants were critical to clearly provide accurate responses; however, not being excellent everywhere, Chinese students were more knowledgeable than foreigners. The difference that we found here may partly come from the language barrier for foreigner to extend the information sources on the studied diseases. In accordance with other previous studies [15, 16, 18, 27], this finding highlighted the need of reinforcing medical student background on HIV/AIDS and STIs especially among foreigners studying in Chinese universities who noticeably showed greater interest to know more about the studied infections.

### 4.2. Attitude and Behavior of Medical Students

Though personal conception can significantly impact individual's behavior, medical students along their education are sufficiently trained and confronted by different illness cases that may offer opportunities to show much positive attitude vis-à-vis all patients regardless of the nature of infection. In this study, forty-five per cent of Chinese medical students compared to thirty-six per cent of foreigners preferred to avoid condom use after HIV testing. Thus, the great proportion would allow unprotected sexual intercourse among unknown HIV status person; however, this study found a lower rate of HIV testing among local students. In fact, several previous studies demonstrated the missed opportunities of medical students and healthcare providers to uptake HIV and STIs testing of populations [6, 13, 19, 27, 28]; and, moreover, many other studies reported that millions of people including medical students with unknown serology are infected with HIV and STIs [29, 30]. So, students personal sexual risk behavior may affect their decision-making towards HIV and associated diseases testing. In this study, though three-fourths of local students never tested for HIV, a significant proportion perceived a link between the development of China and the increase of HIV prevalence. Anyway, a recent study showed an increase of the prevalence of HIV/STIs in China [5]. Perception in our study population could be originated from the unexplained students' beliefs of association between ethnicity and HIV infection. Once again, education and attitude of medical students merit being canalised with accurate and consistent training on HIV/AIDS to overcome the global epidemic.

Although all participants exhibited the willingness to support HIV individuals through several actions, about twenty-nine per cent of Chinese medical students prefer to stay away from classmate or colleague infected with HIV while twenty-four per cent of foreigners though it was unwise to be close to HIV infected person. According to this finding, we suggested that medical student training time period is an opportunity to identify and correct negative attitude leading to discrimination of HIV infected individuals found within participants. More importantly, this study revealed that age, number of sexual partners, HIV testing, considering oneself as high risk individual to HIV, considering physical contact as route of transmission, poverty, and having only books and newspapers as main source of HIV/AIDS information were factors that increased discrimination of people infected with HIV by medical students; however, deeper knowledge subsequently increases medical students' support towards people living with HIV/AIDS and STIs. In accordance with previous studies [8, 15, 17, 31], an overlap misconception, confusion, and fears may lead medical students to discriminate and provide poor medical care towards people infected with HIV and STIs infected individuals. Overall, this study provided substantial evidence of reinforcing education of medical students on HIV/AIDS and STIs with a particular interest in foreign students who are limited in HIV/AIDS information. Therefore, we suggest the introduction of HIV/AIDS and STIs activities through short training, debate, conference, workshops, and symposia in medical student curricula.

### 4.3. Limitations

Several limitations appear to this cross-sectional study in nature. Although all participants were sexually active medical students, they had different background and culture that may affect the disclosure of attitude and behavior. The authors have limited the recruitment of participants to a single university out of numerous universities in China. Therefore, the extension and further clinical observational studies are warranted to assess students' knowledge, attitude, and behavior described in the current study.

## 5. Conclusions

This study has established the overview of knowledge, attitude, and behavior on HIV/AIDS, syphilis, hepatitis B and hepatitis C, and tuberculosis among medical students in Chinese university. Chinese students scored high level of knowledge compared to foreigners while several misconceptions, confusion, and fear existed among the two groups. Students' background and sexual activity strongly affected their attitude and behavior towards people infected with HIV and STIs. The present study arouses much interest among participants and implies the need of reinforcing medical students' education on HIV/AIDS and sexually transmitted infections.

## Figures and Tables

**Table 1:** Participants baseline characteristics.

Variables	Categories	Foreign students (*N* = 157)	Chinese students (*N* = 277)	*p* value
Gender	Male	84 (53.50)	110 (39.71)	0.0055
	Female	73 (46.50)	167 (60.29)	

Median age (IQR)	—	24 (22–26)	25 (23–29)	0.0042

Education level	Bachelor	66 (42.04)	116 (41.88)	0.982
	Master	66 (42.04)	115 (41.52)	
	Doctor	25 (15.92)	46 (16.61)	

Marital status	Married	33 (21.02)	64 (23.10)	<0.001
	Single	77 (49.04)	186 (67.15)	
	Partnered	47 (29.94)	27 (9.75)	

Region of origin	Asia	62 (39.49)	—	—
	Europe	22 (14.01)	—	
	Africa	49 (31.21)	—	
	Others	24 (15.29)	—	

Sexual orientation	Heterosexual	143 (91.08)	249 (90.22)	0.006
	Homosexual	5 (3.18)	23 (8.33)	
	Bisexual	9 (5.73)	4 (1.45)	

Number of sex partners	1	110 (70.06)	240 (86.64)	<0.001
	2	39 (24.84)	34 (12.27)	
	≥3	8 (5.1)	3 (1.08)	

Frequency of condom use during sexual intercourse	Every time	68 (43.31)	135 (48.74)	0.1566
	Often	79 (50.32)	115 (41.52)	
	Never	10 (6.37)	27 (9.75)	

Sex enjoyment with condom	Yes	91 (57.96)	198 (71.48)	<0.0001
	No	64 (40.76)	32 (11.55)	
	Do not know	2 (1.27)	47 (16.97)	

Latest HIV testing	Never tested	12 (7.64)	217 (78.34)	<0.0001
	3 months ago	5 (3.18)	17 (6.14)	
	6 months ago	25 (15.92)	11 (3.97)	
	1 year ago	47 (29.94)	13 (4.69)	
	≥2 years ago	68 (43.31)	19 (6.86)	

Previously tested for STIs and tuberculosis	Syphilis	57 (55.41)	87 (20.58)	<0.0001
	Hepatitis B	101 (73.29)	203 (64.33)	0.0504
	Hepatitis C	93 (59.24)	92 (33.21)	<0.0001
	Tuberculosis	91 (57.96)	68 (24.55)	<0.0001

Previous vaccination	Hepatitis B	102 (79.42)	220 (64.97)	0.0009
	Tuberculosis	94 (52.23)	82 (33.94)	0.0002

*p* < 0.05 is significant; IQR: interquartile range; HIV: human immunodeficiency virus; STIs: sexually transmitted infections.

**Table 2:** Knowledge and sources of information related to HIV/AIDS.

Items	Chinese students	Foreign students	*χ* ^2^/*Z*	*p* value
*Correct answers about HIV/AIDS* ^a^				
The pathogen of HIV/AIDS (virus)	265 (95.67)	78 (49.68)	127.87	<0.0001
The earliest time for HIV positive reaction after exposure (3 months)	76 (27.44)	65 (41.4)	8.91	0.00328
Most people exposed to HIV quickly show serious illness symptoms (No)	266 (96.03)	119 (75.8)	40.96	<0.0001
Reducing the number of sexual partner may protect from HIV/AIDS (Yes)	218 (78.7)	87 (55.41)	26.01	<0.0001
Condom makes sexual relation safe (Agree)	252 (90.97)	54 (34.39)	154.26	<0.0001
Individuals having only one sexual partner can not get HIV or STIs (No)	259 (93.5)	110 (70.06)	43.22	<0.0001
Keeping in good physical condition is the best way to prevent exposure to HIV/AIDS (No)	119 (42.96)	96 (61.15)	13.25	0.0003

*Main sources of information about HIV/AIDS*				
Television or radio	126 (45.49)	110 (70.06)	24.39	<0.0001
School (lectures)	177 (63.9)	106 (67.52)	0.58	0.4472
Internet	145 (52.35)	113 (71.97)	16.01	0.0001
Friends	33 (11.91)	58 (36.94)	37.88	<0.0001
Spouse	25 (9.03)	18 (11.46)	0.67	0.4137
Doctor/nurse	65 (23.47)	53 (33.76)	5.36	0.0206
Books/newspapers/pamphlets and prints	139 (50.18)	82 (52.23)	0.17	0.6816
Other sources	31 (11.19)	22 (14.01)	0.74	0.3884

*Assessment of HIV risky factors*				
Median score^b^ (IQR) of route of transmission	13 (12–14)	13 (12–14)	−1.99	0.0459
Median score^b^ (IQR) of high risk individuals	5 (4–6)	5 (4-5)	−3.54	0.004
Score^b^ of risk factors increasing HIV transmission	4 (3–5)	4 (3–5)	−1.01	0.3130
Previous extracurricular training in HIV and STIs	91 (32.85)	87 (55.41)	21.09	<0.0001
Self-assessment of sufficient knowledge on HIV/AIDS, syphilis, hepatitis, and tuberculosis	63 (22.74)	71 (45.22)	26.617	<0.0001
Students' willingness to know more about HIV	226 (81.59)	140 (89.17)	4.361	0.0368

^a^Only standard and correct answers were reported in this table, and subjected responses were discussed.

^b^Correct answer scored 1 while wrong answer attributed 0.

*p* < 0.05 is significant.

**Table 3:** Attitude and behavior of medical students towards people infected with HIV/AIDS.

	Chinese students	Foreign students	*χ* ^2^/*Z*	*p* value
*Avoidance of condom usage between partners*				
After HIV testing of both partners	126 (45.49)	56 (35.67)	16.27	**0.001**
After mutual decision without HIV testing	47 (16.97)	36 (22.93)		
After marriage	63 (22.74)	22 (14.01)		
Always been used	41 (14.8)	43 (27.39)		
HIV/AIDS infection and ethnicity or culture	210 (75.81)	93 (59.24)	13.07	**0.0003**
Relationship between the development and the increase of HIV in China	1 (1-2)	2 (2-3)	7.54	**<0.0001**
Yes	145 (52.35)	35 (22.29)		
Possible	98 (35.38)	57 (36.31)		
Probably	22 (7.94)	32 (20.38)		
Little/unlikely	5 (1.81)	20 (12.74)		
No	7 (2.53)	13 (8.28)		

*Attitude of students vis-à-vis of friend/classmate infected of HIV/AIDS*				
Inform university	29 (10.47)	10 (6.37)	57.21	**<0.0001**
keep far away from him/her	80 (28.88)	12 (7.64)		
Assist him/her if needed	154 (55.60)	95 (60.51)		
It is not my concern	14 (5.05)	40 (25.48)		
It is unwise to be close to HIV infected person	73 (26.35)	37 (23.57)	0.41	0.5213
People infected of HIV/AIDS should stop attending school or working to be supported by the government and/or NGOs	108 (38.99)	35 (22.29)	12.64	**0.0004**

*Students' attitude towards people living with HIV*				
Must be isolated from other patients	44 (15.88)	45 (28.66)	10.04	**0.0015**
Should be punished by government	11 (3.97)	7 (4.46)	0.06	0.8067
Should be supported by healthcare providers	248 (89.53)	125 (79.62)	8.15	**0.0043**
Should be partnered with HIV negative	11 (3.97)	12 (7.64)	2.70	0.1008
Should be partnered with HIV positive	54 (19.57)	57 (36.31)	14.71	**0.0001**
Should never have a sexual partner	57 (20.65)	28 (17.83)	0.50	0.4779

*Support of students towards HIV/AIDS matters*				
Promote the rights of HIV/AIDS patients	202 (72.92)	112 (71.34)	0.13	0.7225
Fight against stigmatization of HIV/AIDS	205 (74.01)	109 (69.43)	1.05	0.3053
Provide counseling and HIV testing to others	236 (85.2)	125 (79.62)	2.23	0.1353
Educate on safe sex practices	238 (85.92)	141 (89.81)	1.37	0.2420
Promote the behavior change of population	231 (83.39)	124 (78.98)	1.31	0.2523
Provide medical care to HIV/AIDS patients	204 (73.65)	118 (75.16)	0.12	0.7292
Act as peer educator of HIV/AIDS and STIs	226 (81.59)	114 (72.61)	4.76	**0.0291**

*Classification and perception of HIV/AIDS and other surveyed infections: syphilis, hepatitis B, hepatitis C, and tuberculosis (median IQR)*	1 (1–3)	1 (1-2)	−1.40	0.1621
(1) HIV is highly dangerous compared to other infections	236 (54.38)			
(2) HIV is dangerous compared to other infections	92 (21.2)			
(3) HIV is equivalent to other infections	89 (20.51)			
(4) HIV is less dangerous than other infections	17 (3.92)			

*χ* ^2^: Chi-squared; *Z*-value; *p* < 0.05 is significant; IQR: interquartile range; HIV: human immune deficiency virus; AIDS: acquired immunodeficiency syndrome; NGOs: nongovernmental organisations; STIs: sexually transmitted infections.

**Table 4:** Multiple logistic regression analysis of discrimination and support of medical students towards people infected with HIV/AIDS.

Variables	Estimate	SE	OR (95% CI)
*Discrimination of people infected of HIV*			
Chinese students versus foreign students	−2.40	0.55	0.09 (0.03–0.27)
Age	0.16	0.04	1.17 (1.09–1.27)
HIV testing			
Tested 3 months before versus never tested	−1.41	0.63	0.24 (0.07–0.84)
Tested 6 months before versus never tested	0.90	0.51	2.45 (0.91–6.60)
Tested 1 year before versus never tested	−0.10	0.5	0.91 (0.34–2.41)
Tested 2 years or more before versus never tested	−0.09	0.44	0.92 (0.39–2.16)
The number of sexual partners	0.62	0.27	1.85 (1.10–3.12)
Considered medical students under high risk of HIV	0.91	0.45	2.47 (1.01–6.03)
Opposed to physical contact transmission	−1.79	0.48	0.17 (0.07–0.42)
Condoms usage that completely protect against HIV	−1.11	0.36	0.33 (0.16–0.67)
Poverty as factor increasing HIV transmission	−0.84	0.29	0.43 (0.24–0.77)
Already discussed with doctor or nurse HIV/AIDS	−0.59	0.3	0.55 (0.31–1.00)
Reported books/newspaper as main source of information	0.62	0.27	1.86 (1.10–3.17)

*Support to HIV infected individuals*			
Chinese students versus foreign students	1.33	0.5	3.78 (1.41–10.13)
Never been tested for hepatitis B	−1.03	0.36	0.36 (0.18–0.73)
Never been tested for hepatitis C	−0.69	0.32	0.5 (0.27–0.94)
HIV infection increase with biological fluid manipulation	0.65	0.28	1.91 (1.1–3.31)
HIV infected individuals do not show acute symptoms	2.25	0.48	9.47 (3.7–24.27)
Heterosexuals are in high risk of HIV acquisition	−0.67	0.32	0.51 (0.27–0.96)
Bisexuals are under high risk of HIV acquisition	0.59	0.25	1.81 (1.1–2.97)
Lack of awareness could increase HIV transmission	0.66	0.33	1.93 (1.02–3.65)
Poverty could increase HIV transmission	0.79	0.28	2.2 (1.28–3.79)
Acquisition of STIs would increase HIV transmission	−0.94	0.28	0.39 (0.22–0.68)
Tobacco and illicit drugs use could increase transmission	1.41	0.67	4.1 (1.1–15.36)
Condom usage could completely protect against HIV	0.97	0.35	2.62 (1.32–5.24)
Friends as main source of information on HIV/AIDS	0.73	0.36	2.07 (1.02–4.22)
Wanting to know more about HIV/AIDS	1.11	0.31	3.03 (1.66–5.55)

HIV: human immune deficiency virus; AIDS: acquired immunodeficiency syndrome; STIs: sexually transmitted infections; SE: standard error; OR: odds ratio; CI: confident interval.
